# Mechanisims of asthma and allergic disease – 1084. Localization and up-regulation of CysLT2 receptorin perennial allergic rhinitis

**DOI:** 10.1186/1939-4551-6-S1-P80

**Published:** 2013-04-23

**Authors:** Hideaki Shirasaki, Etsuko Kanaizumi, Nobuhiko Seki, Manabu Fujita, Tetsuo Himi

**Affiliations:** 1 Otolaryngology, Sapporo Medical University, Sapporo, Japan; 2 Sapporo Medical University, Japan; 3 Minase Research Institute, Ono Pharmaceutical, Japan

## Background

The cysteinyl leukotrienes (CysLTs) are lipid mediators that have been implicated in the pathogenesis of allergic rhinitis. Pharmacological studies using CysLTs indicate two classes of receptors named CysLT_1_ and CysLT_2_ receptor exist. The former is sensitive to the CysLT_1_ receptor antagonists currently used to treat asthma and allergic rhinitis. We have previously reported the localization of CysLT_1_ receptor by using immunohistochemistry and in situ hybridization. To clarify the expression of CysLT_2_ receptor in human nasal mucosa, we investigated the expression and the localization of CysLT_2_ receptor in human nasal mucosa by means of Western blot analysis and immunohistochemistry.

## Methods

Human turbinates were obtained by turbinectomy from 12 patients with nasal obstruction refractory to medical therapy. CysLT_2_ receptor expression on nasal mucosa was studied by Western blot analysis and immunohistochemistry. Also, to investigate the possible modulation of CysLT_2_ receptor expression, human umbilical vein endothelial cells (HUVECs) were stimulated with IL-4 or IL-13, and CysLT_2_ receptor expression were evaluated by Western blot analysis.

## Results

About 40kDa band was detected in human turbinates by western blot analysis using anti-CysLT_2_ receptor antibody. The expression level of CysLT_2_ receptor protein was marked in patients with nasal allergy than in patients with non-allergic rhinitis. The immunohistochemical study revealed that both vascular endothelial cells and vascular smooth muscles showed intense immunoreactivity for CysLT_2_ receptor. IL-13 enhanced the levels of CysLT2 receptor protein in HUVECs.

## Conclusions

The results suggest a primary role for CysLT_2_ receptor as the vascular responses in upper respiratory tract,and vascular CysLT_2_receptor expression can be regulated by Th2 cytokines.

